# Microbiota characterization of the green mussel *Perna viridis* at the tissue scale and its relationship with the environment

**DOI:** 10.3389/fmicb.2024.1366305

**Published:** 2024-04-11

**Authors:** Liying Chen, Dai Li, Yawei Shen, Zhuo Li, Huanhuan Hao, Caihuan Ke, Zhang Meng, Danqing Feng

**Affiliations:** ^1^State Key Laboratory of Marine Environmental Science, College of Ocean and Earth Sciences, Xiamen University, Xiamen, China; ^2^State Key Laboratory of Mariculture Breeding, College of Ocean and Earth Sciences, Xiamen University, Xiamen, China; ^3^China Nuclear Power Engineering Co., Ltd, Beijing, China

**Keywords:** mussel, *Perna viridis*, tissue-associated microbiota, 16S rRNA gene, microbiota-environment relationship

## Abstract

Research on the microbiota associated with marine invertebrates is important for understanding host physiology and the relationship between the host and the environment. In this study, the microbiota of the green mussel *Perna viridis* was characterized at the tissue scale using 16S rRNA gene high-throughput sequencing and compared with the microbiota of the surrounding environment. Different mussel tissues were sampled, along with two environmental samples (the mussel's attachment substratum and seawater). The results showed that the phyla *Proteobacteria, Bacteroidetes*, and *Spirochaetae* were dominant in mussel tissues. The bacterial community composition at the family level varied among the tissues of *P. viridis*. Although the microbiota of *P. viridis* clearly differed from that of the surrounding seawater, the composition and diversity of the microbial community of the foot and outer shell surface were similar to those of the substratum, indicating their close relationship with the substratum. KEGG prediction analysis indicated that the bacteria harbored by *P. viridis* were enriched in the degradation of aromatic compounds, osmoregulation, and carbohydrate oxidation and fermentation, processes that may be important in *P. viridis* physiology. Our study provides new insights into the tissue-scale characteristics of mussel microbiomes and the intricate connection between mussels and their environment.

## 1 Introduction

Associations between hosts and the microbial communities they harbor are strong in eukaryotes, and thus, they can be viewed as holobionts (Sweet et al., 2011; Pita et al., 2018). A great deal of research has been conducted on the microbiota of marine invertebrates such as sponges and corals, indicating that the microbial associates are involved in the health of marine organisms via functions such as protection against pathogens (Flórez et al., 2015), immune regulation (Eberl, 2010), improved adaptation to environmental changes (Adair and Douglas, 2017; Foster et al., 2017; Koskella et al., 2017), improved nutritional efficiency (Wild et al., 2004, 2009; Naumann et al., 2009), and maintenance of homeostasis (Qin et al., 2010; Tanca et al., 2017). Furthermore, it has been suggested that the microbiota associated with marine invertebrates is distinct from the surrounding environmental bacterial communities, i.e., marine invertebrates harbor selective microorganisms (McFall-Ngai and Ruby, 1991; Thomas et al., 2016; Van Oppen et al., 2018; Nyholm and McFall-Ngai, 2021; Mohamed et al., 2023). Therefore, elucidating the community composition and functional diversity of the microbial community associated with marine invertebrates is important for understanding host physiology and its relationship to the environment.

Mussels are economically important marine invertebrates since many species serve as seafood (Prakoon et al., 2010; Turner et al., 2016; Asaduzzaman et al., 2019). However, mussels can cause economic losses by attachment to marine artificial structures. Mussels use the foot to explore and sense surfaces and secrete proteinaceous byssal threads to attach to the substrates, resulting in serious biofouling problems (Rajagopal et al., 1996; Chavan et al., 2018; Nalini et al., 2019). Meanwhile, mussels have ecological significance, as dense mussel beds provide habitats for other marine species and thereby form ecosystems with high productivity (Hariharan et al., 2021; Lin Z. et al., 2021; Higgins et al., 2022). Furthermore, mussels are often used as bioindicators to monitor coastal environmental quality (Monirith et al., 2003; Li et al., 2013; Leung et al., 2014). As organisms with powerful filter-feeding activity, mussels filter high volumes of seawater from the surrounding environment (Tantanasarit et al., 2013) and consequently accumulate microorganisms in their tissues. To date, most studies concerning the bacteria in mussels have focused on the identification of pathogenic species (Destoumieux-Garzón et al., 2020; Laith et al., 2021; Palamae et al., 2022). Only a few studies have examined the mussel microbiota at the tissue scale and considered the relationship with the environment. For example, recent research on the Manila clam (*Ruditapes philippinarum*) and Mediterranean mussel (*Mytilus galloprovincialis*) found that mussels have tissue-specific microbiota (Meisterhans et al., 2016; Musella et al., 2020), suggesting that the host has a distinct selectivity for microorganisms, a factor that would allow them to function differently in various tissues. However, research focusing on the diversity and functions of the microbiota in mussel tissues is still in its infancy, with most studies reporting the tissue-scale microbiota of the mussels from aquaculture areas or the deep seas (Lin G. et al., 2021; He et al., 2023), and relatively little is known concerning the mussels from natural habitats in coastal environments. Furthermore, most such studies have described the microbiota of the internal tissues, while the microbiota of the tissues exposed to the environment (including the shell, foot, and byssus) is poorly understood.

In this study, we investigated the microbiota of the green mussel *Perna viridis* at the tissue scale and compared the results with the microbiota of the surrounding environment. *P. viridis* is a major species in the coastal waters of Asian tropical and subtropical areas; it is also an invasive species in the Caribbean, North America, and South America (Monirith et al., 2003; Prakoon et al., 2010; Gracia and Rangel-Buitrago, 2020). The green mussels were collected from their natural habitat in the coastal waters of southeast China. Different tissues (mantle, gill, digestive gland, ovary, testis, hemolymph, foot, byssus, and outer shell surface) were sampled, and the attachment substratum of green mussels and the surrounding seawater were also sampled. These samples were analyzed for microbial composition through 16S rRNA gene sequencing. Because it is high-throughput and cost effective, 16S rRNA gene sequencing has been adopted as a mainstream methods for microbiome analysis (Johnson et al., 2019). The objectives of this study were to (1) explore the microbial diversity in different tissues of *P. viridis*, (2) evaluate the relationship of the *P. viridis* microbiota with that of the surrou4nding environment, and (3) analyze the functional profile of microbiota associated with *P. viridis*. This study can provide a comprehensive view of the microbial features at the tissue scale in *P. viridis*, thereby furthering our knowledge of the relationship between mussels and the environment. The findings will also increase our understanding of the role of microbiota in mussel physiology.

## 2 Materials and methods

### 2.1 Sample collection and preparation

**Figure 1:**
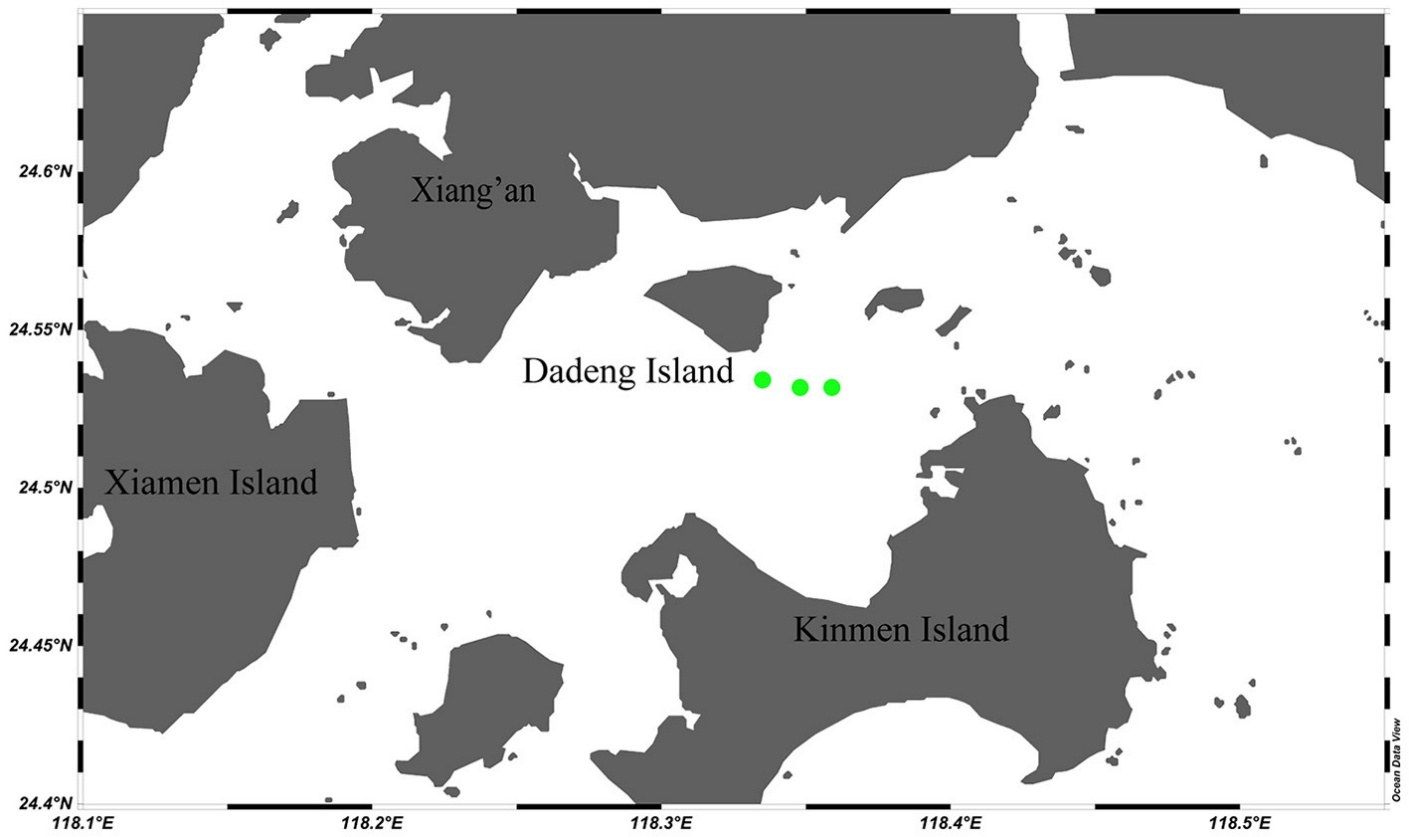
Sampling locations for *Perna viridis* and environmental samples in the Dadeng Sea area (Fujian Province, China). The three green dots represent the three replicate collecting locations for mussels and environmental samples (substratum and habitat seawater).

Sample collection was performed in April 2021 in the area near Dadeng Island (the natural habitat of the green mussel *P. viridis*), Fujian Province, China (Figure 1). Thirty mussel individuals (10 from each of the three sampling sites, 5–7 cm in shell length) were randomly collected and gently washed with 0.1% phosphate-buffered saline (0.1% phosphate buffered saline, PBS). The mussels were immediately stored in coolers (4°C) and transferred to the laboratory within 2 hours. In the laboratory, the mussels were dissected under sterile conditions. The adductor muscle of each green mussel individual was incised with a sterile scalpel. The hemolymph (H) was then taken from the posterior adductor muscle using a sterile 1 ml syringe, transferred to sterile tubes, and immediately frozen in liquid nitrogen following the method of Musella et al. (2020). The byssus (B), mantle (M), gill (G), foot (F), digestive gland (D), and gonad (testis, T; ovary, O) were dissected from each green mussel individual using a sterile scalpel, rinsed with sterile PBS, transferred to sterile tubes, and immediately frozen in liquid nitrogen. All the samples were stored at −80°C.

The samples for the outer shell surface (U) and substratum (S) of *P. viridis* were obtained by swab sampling. Swab samples were taken by washing the outer shell surface and the substratum (nylon rope) with 0.1% PBS and then gently rubbing the surfaces using cotton swabs. The obtained swab samples were stored in sterile tubes at 4°C and then transferred to a −80°C refrigerator. A total of 1.5 L of seawater (W) was also collected from each of the three sampling sites (Figure 1). Seawater samples were stored in coolers (4°C) and transferred to the laboratory along with the mussel and swab samples. In the laboratory, the seawater samples were filtered with MF-Millipore membranes (0.22 μm, Biosharp, Anhui, China). The membranes were then immediately frozen in liquid nitrogen and stored at −80°C.

### 2.2 Microbial DNA extraction

Total microbial DNA was extracted from the tissue and environmental samples using a HiPure Stool DNA Kit (Magen, Guangzhou, China) according to the manufacturer's protocol. Specifically, 200 μl of hemolymph, 20–30 mg of byssus, mantle, gill, foot, digestive gland, testis, or ovary, one filtered seawater sample, and two to three cotton swabs of the outer shell surface or substratum were used for total microbial DNA extraction. DNA concentrations were measured using a Qubit^®^ dsDNA HS Assay Kit (Thermo Fisher Scientific, Waltham, MA, USA).

### 2.3 Library construction and sequencing

The library construction and next-generation sequencing (NGS) were conducted by GENEWIZ (Suzhou, China). The sequencing library was constructed using a MetaVX Library Preparation Kit (GENEWIZ, Suzhou, China). Briefly, 20–30 ng of DNA was used to generate amplicons that covered the V3–V4 hypervariable regions of the bacterial 16s rRNA gene. The forward primer sequence was 5′-CCTACGGRRBGCASCAGKVRVGAAT-3′, and the reverse primer sequence was 5′-GGACTACNVGGGTWTCTAATCC-3′. The concentration was detected using a microplate reader (Tecan, Infinite 200 Pro), and the fragment sizes (expected value ~600 bp) were measured through 1.5% agarose gel electrophoresis.

The PCR amplification procedure was as follows. A 25 μl of PCR mixture was prepared containing 2.5 μl of TransStart buffer, 2 μl of dNTPs (2.5 mM each), 1 μl of forward primer, 1 μl of reverse primer, 0.5 μl of TransStart Taq DNA polymerase, and 17.5 μl of gDNA. The first round PCR was performed by the following program: 3 min of denaturation at 94°C followed by 14–16 cycles of denaturation at 94°C for 10 s, annealing at 57°C for 90 s, elongation at 72°C for 15 s, and a final extension at 72°C for 5 min. In addition, a splice with Index primers was added to the end of the PCR product of 16S rDNA by PCR to facilitate NGS sequencing. The 50 μl of PCR mixture was prepared containing 2.5 μl of TransStart buffer, 2 μl of dNTPs (2.5 mM each), 3 μl of INDEX primer N, 3 μl of Index primer S, 4 μl of 1x cocktail, 0.5 μl of TransStart Taq DNA polymerase, and 10 μl of ddH_2_O. The second round of the PCR procedure was performed using the following program: 3 min of denaturation at 94°C followed by 10–12 cycles of denaturation at 94°C for 10 s, annealing at 60°C for 30 s, elongation at 72°C for 15 s, and a final extension at 72°C for 5 min at 4°C.

The libraries were quantified to a final concentration of 10 nM. Sequencing was performed on a MiSeq platform (Illumina, San Diego, CA, USA) using a 300 paired-end protocol according to the instrument manual. The MiSeq Control Software (MCS) was used to obtain raw sequencing data.

### 2.4 Bioinformatic analyses

Raw reads were processed with the software Qiime (1.9.1) (Caporaso et al., 2010) to splice forward and reverse reads according to their overlap. Data with low quality were then removed from the spliced sequences using Cutadapt (1.9.1) (Martin, 2011), and chimeras were removed with Vsearch (1.9.6) (Rognes et al., 2016).

After quality filtering, an average of 84,710 reads per sample were clustered into operational taxonomical units (OTUs) using a 97% similarity threshold criterion. After normalization of the entire dataset, all 68,306 remaining OTU sequences were searched against the Silva 138 16SrRNA database (http://www.arb-silva.de/). Finally, the species taxonomic analysis of the representative OTU sequences was completed by using the Bayes algorithm of the Ribosomal Database Program classifier, and the community composition of each sample was examined at several taxonomic levels.

Alpha diversity indices (the Chao1 and Shannon indexes) were estimated through sequence random sampling according to the effective sequence number and calculated using Qiime (1.9.1). Rank-abundance was constructed in R (v 3.3.1) based on the OTU analysis results. Beta diversity was estimated by unweighted UniFrac analysis and principal coordinates analysis (PCoA). Unweighted UniFrac distances were calculated using Qiime (1.9.1), and the significance of data separation in the PCoA was based on the distances in the Bray-Curtis matrix. Finally, the functional profile analysis of the microbial community in each sample was performed using the software PICRUSt (v1.0.0) against the Kyoto Encyclopedia of Genes and Genomes (KEGG) categories.

### 2.5 Statistical analyses

Values were expressed as the mean ± standard deviation. The differences in alpha diversity among samples were statistically analyzed through Kruskal–Wallis tests, and a *p* < 0.05 was defined as indicating statistical significance.

## 3 Results

### 3.1 Microbiota profiling of *P. viridis* and environmental samples

**Figure 2:**
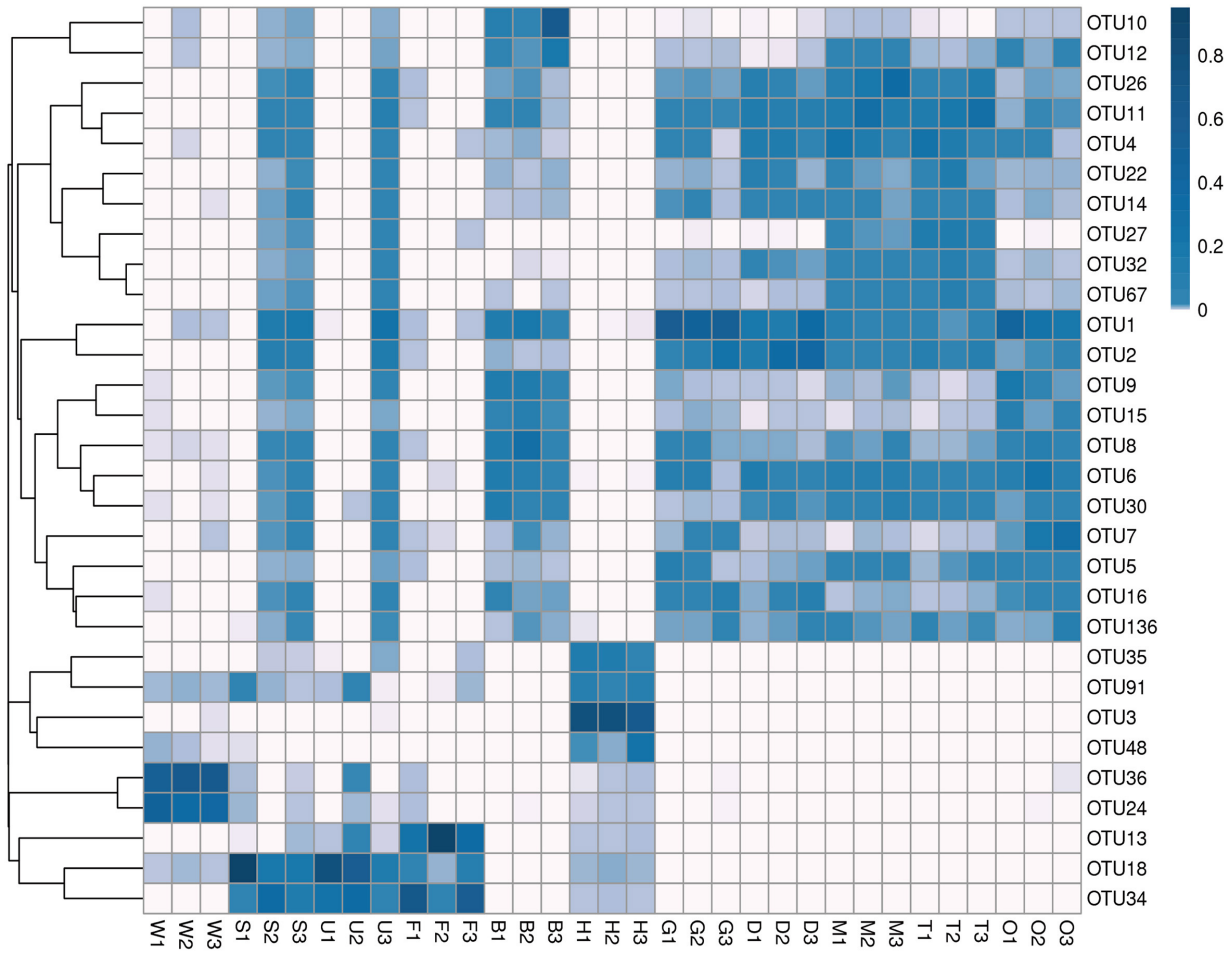
Cluster heatmap of the top 30 OTUs in mussel tissues and environmental samples. Samples are shown column-wise, and OTUs are shown row-wise. The heatmap indicates the normalized relative OTU abundance clustered by OTUs (rows). W, seawater; S, substratum; U, outer shell surface; F, foot; B, byssus; H, hemolymph; D, digestive gland; G, gill; M, mantle; T, testis; O, ovary.

Microbial communities were sequenced from 33 samples [27 tissue samples, including three each from hemolymph, byssus, mantle, gill, foot, digestive gland, ovary, testis, and outer shell surface samples, and six environmental samples (three substratum and three seawater samples)]. The NGS was conducted on the V3–V4 hypervariable region of the 16S rRNA gene to obtain the microbial composition and structure of each sample. The sequencing runs generated a total of 2,795,441 paired-end reads (46,806–132,794 reads per sample; Supplementary Table S2). A total of 2,254,088 clean reads passed quality filtering (40,762–100,570 per sample; Supplementary Table S2) and were clustered into 1,951,958 OTUs. According to the heatmap of the 30 most abundant OTUs (Figure 2), the samples could be divided into four clusters. The first cluster comprised samples from seawater; the second was composed of foot, outer shell surface, and substratum; the third comprised byssus, digestive gland, gill, mantle, ovary, and testis; and the fourth was the hemolymph, with specific OTUs that were rare in other samples.

**Figure 3:**
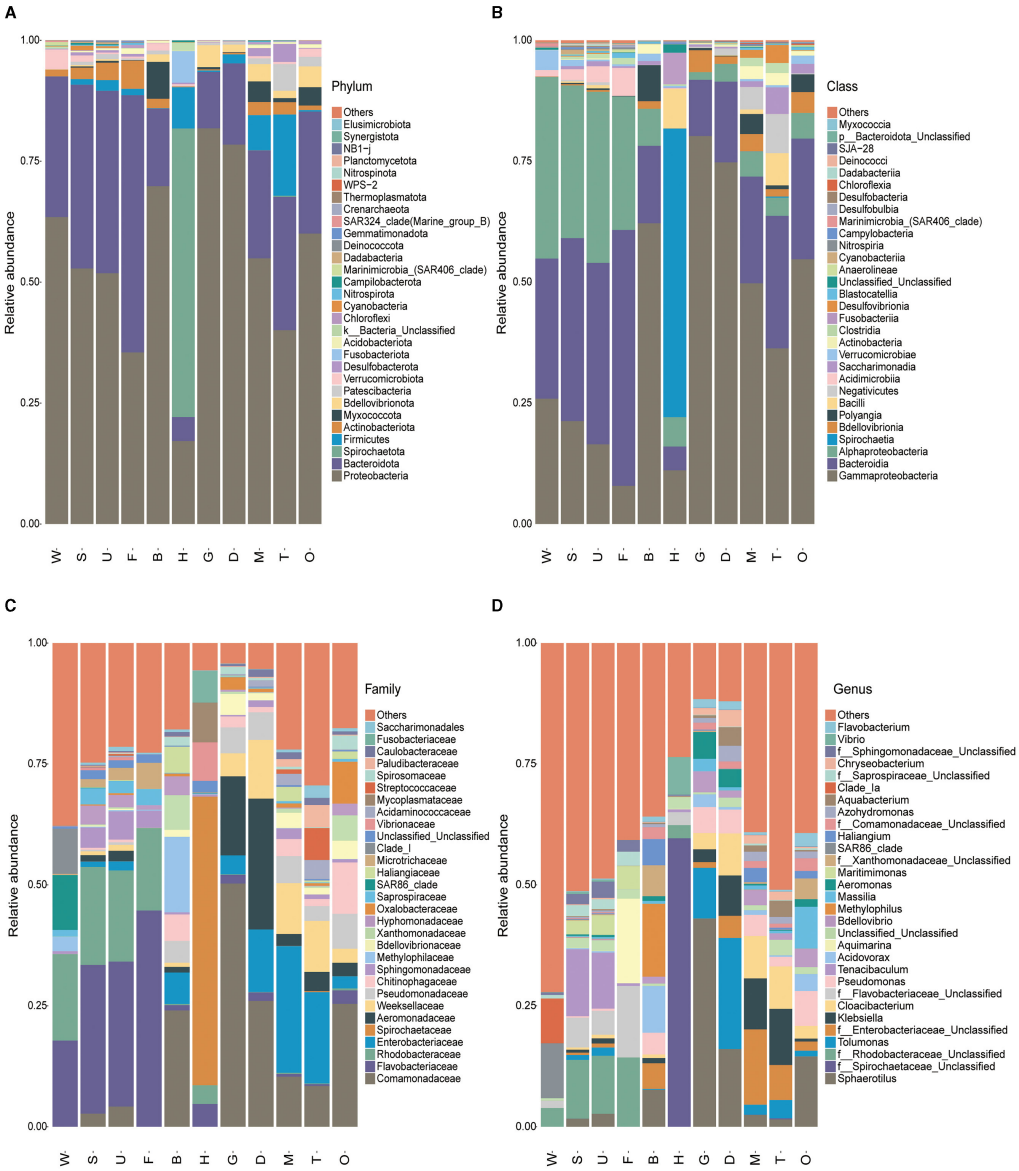
Overall composition of the microbiota in mussel tissues and environmental samples. The bar charts show the distribution of the top 30 microbial species in 11 samples at **(A)** phylum, **(B)** class, **(C)** family, and **(D)** genus levels. W, seawater; S, substratum; U, outer shell surface; F, foot; B, byssus; H, hemolymph; D, digestive gland; G, gill; M, mantle; T, testis; O, ovary.

Amplicon sequencing revealed the overall microbial composition of *P. viridis* tissues (Figure 3). At the phylum level, *Proteobacteria* (mean relative abundance (r.a.) ± SD, 54.40 ± 20.94%), *Bacteroidetes* (23.94 ± 14.94%), and *Spirochaetae* (6.66 ± 19.20%) were dominant in mussel tissues, followed by *Firmicutes* that accounted for 4.30% (Figure 3A). In addition, phylogenetic trees of the top 30 genera showed that most of the microorganisms belonged to *Proteobacteria* and *Bacteroidetes* (Supplementary Figure S2). Overall, the bacterial community composition at the phylum level was similar among different mussel tissues, except for hemolymph that was dominated by *Spirochaetae*. At the class level, *Gammaproteobacteria* (43.72 ± 26.90%), *Bacteroidia* (23.79 ± 14.88%), and *Alphaproteobacteria* (10.69 ± 12.10%) were dominant in mussel tissues (Figure 3B). At the family level, the most representative families were *Comamonadaceae* (16.54 ± 16.64%), *Flavobacteriaceae* (9.75 ± 16.51%), *Enterobacteriaceae* (8.09 ± 9.65%), and *Spirochaetaceae* (6.66 ± 19.20%) (Figure 3C and Supplementary Table S3). At the genus level, the dominant genera were *Sphaerotilus* (9.78 ± 13.81%), the unclassified genus of *Spirochaetaceae* (6.66 ± 19.20%), and *Tolumonas* (4.71% ± 8.29%) (Figure 3D).

Regarding the microbial community composition of environmental samples, *Proteobacteria* (58.15 ± 7.88%) and *Bacteroidetes* (33.53 ± 7.78%) were the dominant phyla (Figure 3A). At the class level, *Alphaproteobacteria* (34.54 ± 8.10%), *Bacteroidia* (33.39 ± 7.77%), and *Gammaproteobacteria* (23.60 ± 6.03%) were dominant in environmental samples (Figure 3B). The most representative families were *Flavobacteraceae* (24.27 ± 9.65%) and *Rhodobacteraceae* (19.08 ± 5.63%) (Figure 3C). The dominant genera were the unclassified genera of *Rhodobacteraceae* (8.01 ± 5.44%) and *Tenacibaculum* (6.94 ± 11.35%) (Figure 3D).

The bacterial community composition at the family level differed among the mussel tissues (Figure 3C and Supplementary Table S3). *Spirochaetaceae, Vibrionaceae*, and *Mycoplasmataceae* were the dominant bacteria in hemolymph, while the representative families in the mantle, digestive gland, gill, and gonad were *Comamonadaceae, Enterobacteriaceae, Aeromonadaceae*, and *Weeksellaceae*. *Comamonadaceae* and *Methylophilaceae* were dominant in mussel byssus. It was interesting to note that the foot and outer shell surface had similar dominant bacteria (*Flavobacteriaceae* and *Rhodobacteraceae*) with the substratum. These results implied that the microbiota of the tissues exposed or partly exposed to the environment (the outer shell surface and foot) was distinct from that of the tissues inside the shells. In particular, the foot (the organ responsible for exploring the substratum), the outer shell surface (the part exposed to the surrounding environment), the substratum, and seawater all shared the same dominant taxa (i.e., *Flavobacteriaceae* and *Rhodobacteraceae*), indicating the close connection of microbiota of foot and shells with that of the surrounding environment.

### 3.2 Microbial diversity analyses of *P. viridis* and environmental samples

**Figure 4:**
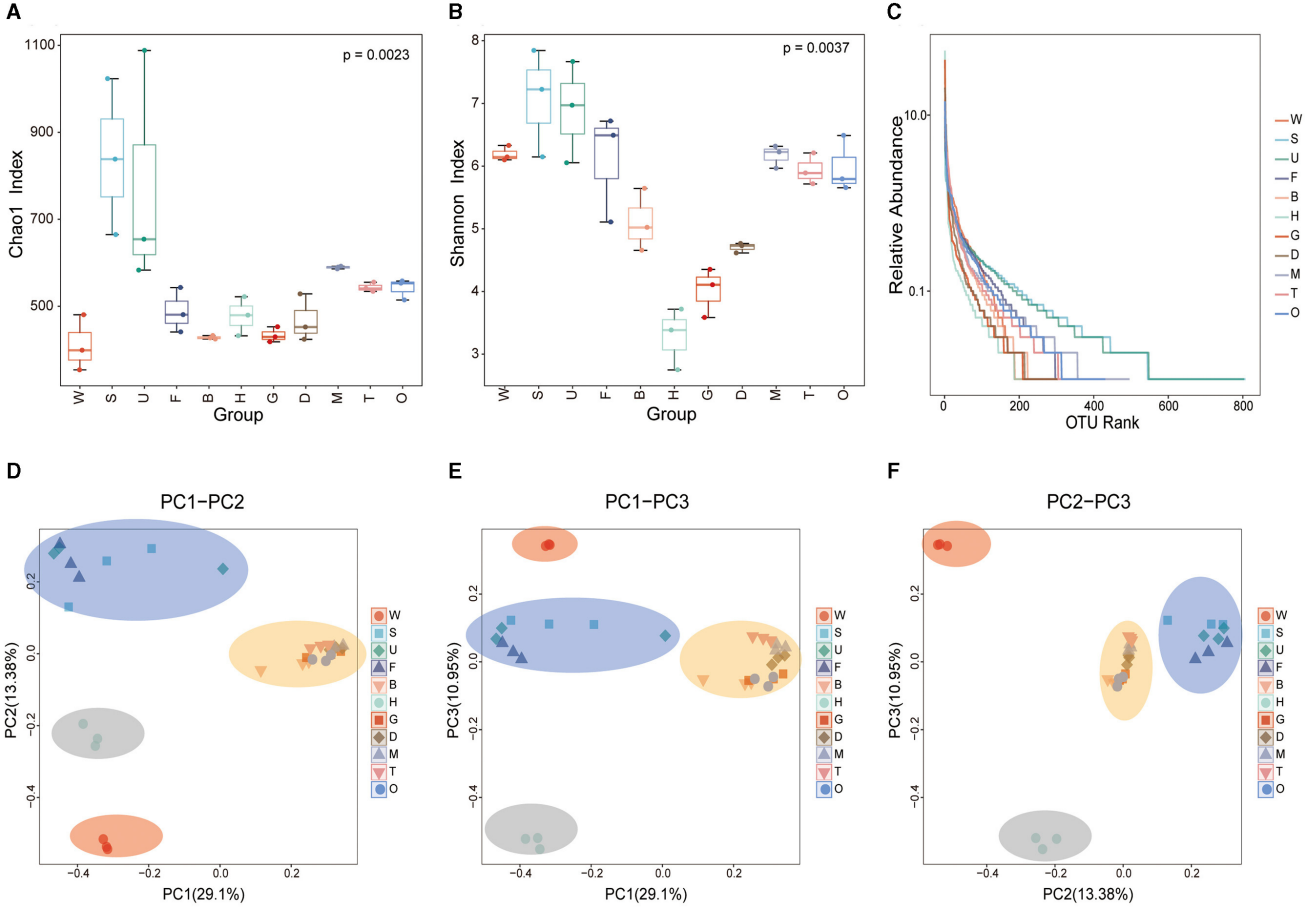
Alpha and beta diversity of the microbiota in mussel tissues and environmental samples. Box and whisker plots show the results of alpha diversity, including **(A)** the Chao1 index; **(B)** the Shannon diversity index; and **(C)** rank-abundance reflecting both species abundance (the length on the horizontal axis) and species evenness (the smoothness of the curve). **(D–F)** Beta diversity based on unweighted UniFrac distances between the microbiota structures of different samples. PCoA mapping analysis was performed based on the Bray-Curtis distance matrix. W, seawater; S, substratum; U, outer shell surface; F, foot; B, byssus; H, hemolymph; D, digestive gland; G, gill; M, mantle; T, testis; O, ovary.

Alpha diversity analysis results showed that species richness in mussel tissues was significantly different from the diversity in the environmental samples (Kruskal–Wallis test, *p* < 0.05). According to the Chao1 index which evaluates the total number of species in a sample, the total number of microbial species was much higher in the substratum samples than in other samples, with the species number in the seawater sample being the lowest (Figure 4A). Among mussel tissues, the outer shell surface had the highest microbial diversity. In contrast, the microbial diversity of the byssus was the lowest. The Shannon diversity index (Figure 4B) showed that the substratum had the highest microbial diversity, while hemolymph had the lowest. The same trend was seen in the rank-abundance curve, where the abundance and evenness of microbial species in the substratum were the highest, and the hemolymph was the lowest (Figure 4C).

For the beta diversity analysis, a Bray-Curtis PCoA was performed to explore the microbial composition in different mussel tissues and environmental samples. The mussel tissues and environmental samples could be divided into four categories (Figures 4D–F). The microbial communities in the samples of the foot, outer shell surface, and substratum were similar. The seawater sample comprised a separate category. Byssus, gill, digestive gland, mantle, ovary, and testis were classified as one category, while hemolymph clustered apart from all these tissues owing to its unique microbial composition. Interestingly, the microbial diversity in the foot and outer shell surface was closer to that of the substratum rather than to seawater, suggesting that the mussel foot and shell have microbial connections with the environment, especially the substratum.

### 3.3 KEGG functional prediction of *P. viridis* and environmental microbiome

**Figure 5:**
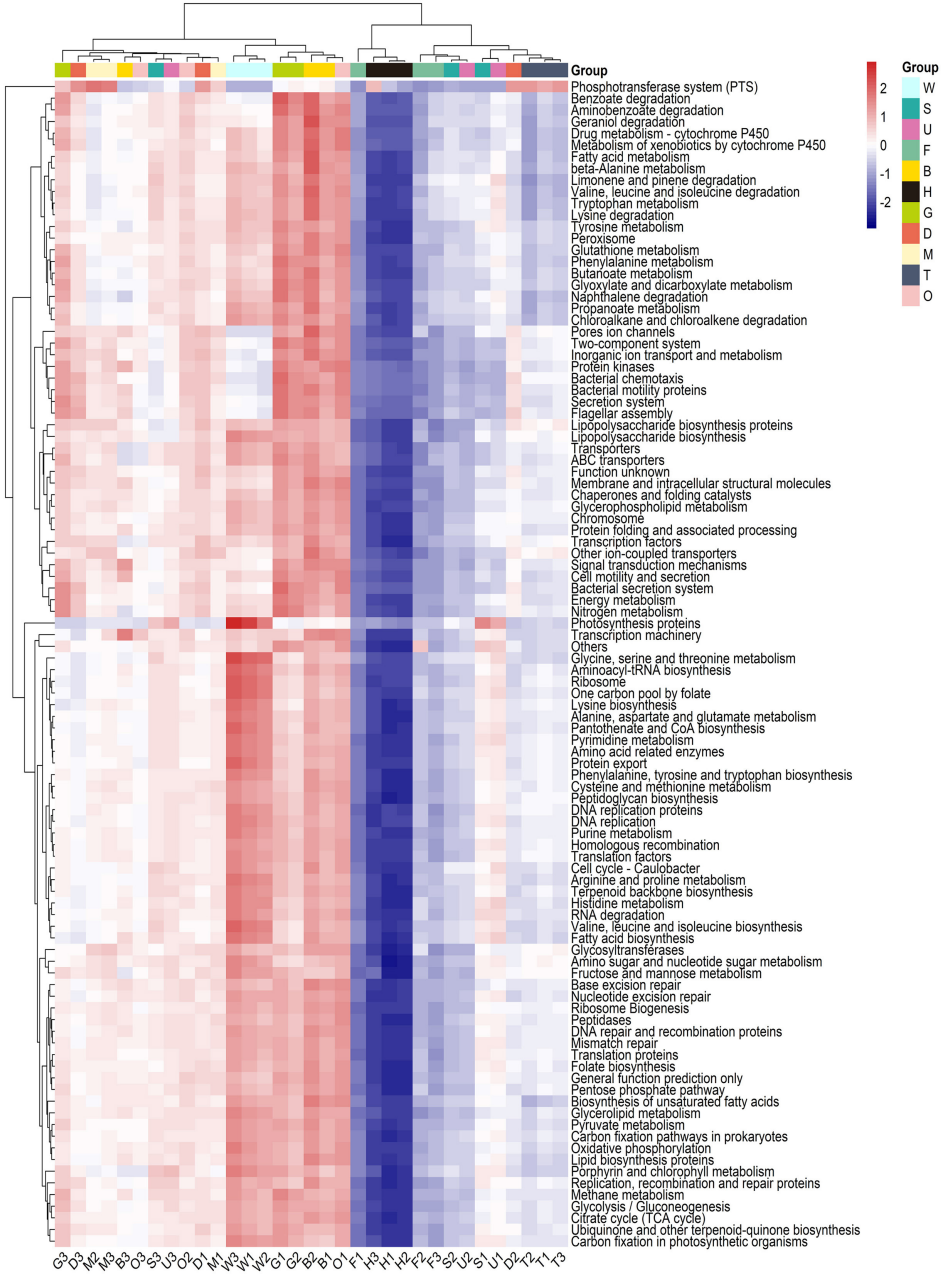
KEGG function prediction analysis of mussel tissues and environmental samples. The heatmap was based on the top 100 abundant pathways sorted by median difference. Samples are shown column-wise, and functional categories (metabolic pathways in the KEGG database) are shown row-wise. The boxes on the top of each column are colored according to their sample type. W, seawater; S, substratum; U, outer shell surface; F, foot; B, byssus; H, hemolymph; D, digestive gland; G, gill; M, mantle; T, testis; O, ovary.

The functional profiles of the microbial communities for tissues and environmental samples were predicted using PICRUSt based on phylogenetic information. A total of 15,039 KOs (KEGG Orthology) were obtained through PICRUSt function prediction analysis, and 296 pathways were annotated. Pathways with high abundance included transporters, general function prediction only, and ABC transporters pathways, while pathways with low abundance included the biosynthesis of type II polyketide products and the biosynthesis of 12-, 14-, and 16-membered macrolides. The top 100 pathways ranked by abundance were utilized to cluster all 33 samples (Figure 5). The samples could be divided into two groups. In group I, seawater was clustered with the gill, byssus, digestive gland, mantle, and ovary. In group II, the foot, outer shell surface, substratum, testis, and hemolymph were clustered, with the hemolymph isolated from the other samples. As shown in Figure 5, most pathways exhibited a higher relative abundance in group I than in group II. For specific samples, the hemolymph microbiome was characterized by the pathways involved in osmotic regulation (taurine and hypotaurine metabolism), polycyclic aromatic hydrocarbon degradation, purine metabolism, and other environmental information processing. The outer shell surface and foot samples were clustered with substratum, and they were enriched in the degradation of aromatic compounds such as limonene and pinene degradation, RNA degradation, and glycosyltransferases. The byssus and gill microbiomes were characterized by the pathways involved in benzoate degradation, geraniol degradation, and fatty acid metabolism. The results showed a specific profile of seawater characterized by the enrichment in pathways involved in photosynthesis protein and nitrogen cycle (including glycine, serine, and glutamate metabolism and arginine and proline metabolism).

## 4 Discussion

In this study, we characterized the microbial composition in the green mussel *P. viridis* at the tissue scale and compared the results with the microbial composition of the surrounding environment. The results from this study revealed that the microbiota associated with the green mussel was well differentiated from that of the surrounding environment, but there was also some association between the two.

We found that *Proteobacteria* and *Bacteroidetes* were the dominant phyla in both mussel tissues and environmental samples. *Proteobacteria* constitute one of the most diverse microbial phyla on Earth, possessing a wide range of metabolic characteristics (Spain et al., 2009; Zhou et al., 2020). For example, it has been suggested that the proteobacterial endosymbionts of bivalves play a vital role in the oxidation of reduced sulfur, methane, hydrogen, and carbon monoxide (Duperron et al., 2007). At the family level, there were remarkable difference between the tissues and environmental samples. The *Comamonadaceae, Flavobacteriaceae, Enterobacteriaceae*, and *Spirochaetaceae* families were dominant in the tissues of *P. viridis*. Among these, *Comamonadaceae* has been reported to be involved in hydrooxidation (Brazelton et al., 2012), denitrification, iron reduction, primary production in low-nutrient underground environments (Deja-Sikora et al., 2019), and pollution removal (Cui et al., 2017). Researchers have found that *Enterobacteriaceae* was the dominant family in two deep-sea mussels, *Bathymodiolus platifrons* and *B. japonicas*, from cold spring and hydrothermal environments, and that it may be involved in element cycling (Lin G. et al., 2021). Here, we observed their dominance in the tissues of mussels from a coastal environment. At the genus level, *Sphaerotilus* was the most dominant genus in the tissues of *P. viridis. Sphaerotilus* is well-known for its iron-oxidizing activity, and it contributes to the biogeochemical cycling of nitrogen and iron (Hoeniger et al., 1973). Our findings indicate that most bacteria of the genus *Sphaerotilus* were associated with the gill and digestive gland in *P. viridis*.

Bivalves have open-vessel circulatory systems, and the hemolymph usually reflects the host condition (Lokmer and Mathias Wegner, 2015). Our data showed that the dominant microbial families in the hemolymph of *P. viridis* were *Spirochaetaceae, Vibrionaceae* and *Mycoplasmataceae*. The families *Spirochaetaceae* and *Mycoplasmataceae* were recently identified as core members of the Manila clam (*Ruditapes philippinarum*) and Pacific oyster (*Crassostrea gigas*) microbiota (Milan et al., 2018; King et al., 2020; Offret et al., 2020). Most *Vibrionaceae* strains are pathogenic bacteria. For example, *Vibrio splendidus* infection can induce dysbiosis in the blue mussel *M. edulis* (Ben Cheikh and Travers, 2022). The microorganisms in bivalve hemolymph are mediated by the host. Researchers found that after the antimicrobial peptides were silenced by RNA interference, γ-proteobacteria increased significantly, and *Vibrio* sp. proliferated in the hemolymph of scallops (González et al., 2020). How mussels mediate the microorganisms in hemolymph needs further study.

Mussels attach themselves to substrates via the byssus, a protein anchor composed of threads and sticky plaques (Von Byern and Grunwald, 2010). A genome-wide transcriptome analysis of *P. viridis* revealed that to defend byssus proteins from proteases and bacteria, the mussel foot secretes defense proteins, including lysozyme, proteinase inhibitors, and possibly lectins (Inoue et al., 2021). In this study, the Chao1 index indicated that the mussel byssus had the lowest number of microbiota species among mussel tissues, a result that may be due to the defense proteins mentioned earlier.

The results of the alpha diversity analysis showed that the total numbers of microbial species and diversity of the substratum samples were significantly higher than those of mussel tissues. The low microbial diversity in mussel tissues may be because microorganisms in the internal tissues of mussels have been screened, colonized, or enriched (Musella et al., 2020; Inoue et al., 2021; He et al., 2023). Interestingly, the dominant microbiota of the foot and outer shell surface was the same as that of the substratum. The results of the PCoA also revealed that the microbial samples from the foot, outer shell surface, and substratum were clustered in one category, indicating that they harbor similar microbial communities. It seems reasonable that the outer shell surface and substratum of *P. viridis* have similar microbial communities since they are exposed to the same microenvironment. The findings in this study have demonstrated the close connection of the foot microbiota with the microbiota of the outer shell surface and substratum, a pattern that may be due to the exploration behavior of the foot. The mussel extends its foot to explore suitable substrate before secreting the byssus (Yonge, 1962). During exploration, the foot actively contacts the substrate and the outer shell surfaces of other mussels, possibly resulting in the similarity of the microbiota of the foot with that of the surrounding surfaces.

The results of KEGG function prediction showed that the most abundant pathways in tissue samples included the transporters, general function prediction only, and ABC transporters pathways, which were also the most abundant pathways in environmental samples. Pathways related to transporters and ABC transporters are involved in many vital biological processes (Locher, 2016; Thomas et al., 2020). The least abundant pathway in environmental samples was the biosynthesis of type II polyketide products, while in tissue samples, the least abundant pathway was the biosynthesis of 12-, 14-, and 16-membered macrolides. Both pathways were involved in the synthesis of specific secondary metabolites. Furthermore, the KEGG results indicated distinct functional patterns between the microbiota in the environment and mussel tissues. The environmental microorganisms were involved in the sulfur and nitrogen cycles, including histidine metabolism, isoquinoline alkaloid biosynthesis, and nitrogen transformation. The mussel tissues microbiota was enriched in the degradation of aromatic compounds, osmoregulation, and carbohydrate oxidation or fermentation, functions that may benefit the health of mussels. For example, it has been reported that polycyclic aromatic hydrocarbons can reduce the stability of lysosomal membranes and filtration efficiency in mussels (Karacik et al., 2009). It is reasonable to speculate that the degradation of such aromatic compounds by tissue microbiota may play a role in the maintenance of homeostasis and the adaptation to environmental pollution in mussels.

## 5 Conclusions

In the present study, the tissue-associated microbial community structure of the green mussel *P. viridis* collected from natural coastal habitats was characterized and compared with that of its surrounding environment. The results confirmed the variation in microbiota at the tissue scale in *P. viridis*. Although the microbiota of *P. viridis* clearly differed from the seawater microbiota, the composition and diversity of the microbial community of its foot and outer shell surface were similar to those of the substratum. This finding provides new insights into the strong connection between mussels and their environment. Functional predictions revealed that the bacteria harbored by *P. viridis* were involved in the degradation of aromatic compounds, osmoregulation, and carbohydrate oxidation or fermentation processes that may play roles in the maintenance of health in *P. viridis*. Future work can focus on the possible mechanisms that control bacterial-host interactions in mussels and the specific effects of the tissue-associated bacteria on the physiology of mussels.

## Data availability statement

The data presented in this study are publicly available. The data presented in the study are deposited in the NCBI SRA repository (https://www.ncbi.nlm.nih.gov/sra), accession number PRJNA1085882.

## Author contributions

LC: Conceptualization, Investigation, Methodology, Software, Writing – original draft, Writing – review & editing. DL: Data curation, Software, Writing – review & editing. YS: Formal analysis, Project administration, Writing – original draft. ZL: Data curation, Investigation, Methodology, Writing – original draft. HH: Formal analysis, Project administration, Writing – original draft, Supervision. CK: Conceptualization, Formal analysis, Resources, Writing – review & editing. ZM: Conceptualization, Funding acquisition, Resources, Writing – review & editing. DF: Conceptualization, Funding acquisition, Resources, Writing – review & editing.

## References

[B1] Adair K. L. Douglas A. E. (2017). Making a microbiome: the many determinants of host-associated microbial community composition. Curr. Opin. Microbiol. 35, 23–29. 10.1016/j.mib.2016.11.002 27907842

[B2] Asaduzzaman M. Rahi Noor A. Rahman M. M. Akter S. Hoque N. F. Shakil A.. (2019). Reproductive biology and ecology of the green mussel *Perna viridis*: a multidisciplinary approach. Biology 8:88. 10.3390/biology8040088 31731653 PMC6955735

[B3] Ben Cheikh Y. Travers M. A. (2022). *Vibrio splendidus* infection induces dysbiosis in the blue mussel and favors pathobiontic bacteria. Microbiol. Res. 261, 127078. 10.1016/j.micres.2022.127078 35640531

[B4] Brazelton W. J. Nelson B. Schrenk M. O. (2012). Metagenomic evidence for H_2_ oxidation and H_2_ production by serpentinite-hosted subsurface microbial communities. Front. Microbiol. 2:268. 10.3389/fmicb.2011.00268 22232619 PMC3252642

[B5] Caporaso J. G. Kuczynski J. Stombaugh J. Bittinger K. Bushman F. D. Costello E. K.. (2010). QIIME allows analysis of high-throughput community sequencing data. *Nat. Methods* 7, 335–336. 10.1038/nmeth.f.303 20383131 PMC3156573

[B6] Chavan P. Kumar R. Joshi H. Kirubagaran R. Venugopalan V. P. (2018). Multimarker study of the effects of antifouling biocide on benthic organisms: results using *Perna viridis* as candidate species. Environ. Sci. Pollut. Res. 25, 20407–20418. 10.1007/s11356-017-9607-z 28685340

[B7] Cui M. Zhang W. Fang J. Liang Q. Liu D. (2017). Carbon and hydrogen isotope fractionation during aerobic biodegradation of quinoline and 3-methylquinoline. Appl. Microbiol Biotechnol. 101, 6563–6572. 10.1007/s00253-017-8379-1 28623382

[B8] Deja-Sikora E. Gołebiewski M. Kalwasińska A. Krawiec A. Kosobucki P. Walczak M.. (2019). *Comamonadaceae* OTU as a remnant of an ancient microbial community in Sulfidic waters. Microb. Ecol. 78, 85–101. 10.1007/s00248-018-1270-5 30341500 PMC6560000

[B9] Destoumieux-Garzón D. Canesi L. Oyanedel D. Travers M. Charrière G. M. Pruzzo C.. (2020). *Vibrio*–bivalve interactions in health and disease. Environ. Microbiol. 22, 4323–4341. 10.1111/1462-2920.15055 32363732

[B10] Duperron S. Sibuet M. MacGregor B. J. Kuypers M. M. M. Fisher C. R. Dubilier N.. (2007). Diversity, relative abundance and metabolic potential of bacterial endosymbionts in three *Bathymodiolus* mussel species from cold seeps in the Gulf of Mexico. Environ. Microbiol. 9, 1423–1438. 10.1111/j.1462-2920.2007.01259.x 17504480

[B11] Eberl G. (2010). A new vision of immunity: homeostasis of the superorganism. Mucosal Immunol. 3, 450–460. 10.1038/mi.2010.20 20445502

[B12] Flórez L. V. Biedermann P. H. W. Engl T. Kaltenpoth M. (2015). Defensive symbioses of animals with prokaryotic and eukaryotic microorganisms. Nat. Prod. Rep. 32, 904–936. 10.1039/C5NP00010F 25891201

[B13] Foster K. R. Schluter J. Coyte K. Z. Rakoff-Nahoum S. (2017). The evolution of the host microbiome as an ecosystem on a leash. Nature 548, 43–51. 10.1038/nature23292 28770836 PMC5749636

[B14] González R. Gonçalves A. T. Rojas R. Brokordt K. Rosa R. D. Schmitt P.. (2020). Host defense effectors expressed by hemocytes shape the bacterial microbiota from the scallop hemolymph. Front. Immunol. 11:599625. 10.3389/fimmu.2020.599625 33281827 PMC7689009

[B15] Gracia C. A. Rangel-Buitrago N. (2020). The invasive species *Perna viridis* (Linnaeus, 1758 - Bivalvia: *Mytilidae*) on artificial substrates: a baseline assessment for the Colombian Caribbean Sea. Marine Pollut. Bullet. 152:110926. 10.1016/j.marpolbul.2020.110926 32479298

[B16] Hariharan G. Purvaja R. Anandavelu I. Robin R. S. Ramesh R. (2021). Accumulation and ecotoxicological risk of weathered polyethylene (wPE) microplastics on green mussel (*Perna viridis*). *Ecotoxicol. Environ. Safety* 208:111765. 10.1016/j.ecoenv.2020.111765 33396084

[B17] He J. Jia M. Wang J. Wu Z. Shao S. He Y.. (2023). Mytilus farming drives higher local bacterial diversity and facilitates the accumulation of aerobic anoxygenic photoheterotrophic related genera. Sci. Total Environ. 856:158861. 10.1016/j.scitotenv.2022.158861 36419274

[B18] Higgins E. Parr T. B. Vaughn C. C. (2022). Mussels and local conditions interact to influence microbial communities in mussel beds. Front. Microbiol. 12:790554. 10.3389/fmicb.2021.790554 35095802 PMC8793333

[B19] Hoeniger J. F. Tauschel H. D. Stokes J. L. (1973). The fine structure of *Sphaerotilus natans*. Can. J. Microbiol. 19, 309–313. 10.1139/m73-051 4121377

[B20] Inoue K. Yoshioka Y. Tanaka H. Kinjo A. Sassa M. Ueda I.. (2021). Genomics and transcriptomics of the green mussel explain the durability of its byssus. Sci. Rep. 11:5992. 10.1038/s41598-021-84948-6 33727571 PMC7971044

[B21] Johnson J. S. Spakowicz D. J. Hong B.-Y. Petersen L. M. Demkowicz P. Chen L.. (2019). Evaluation of 16S rRNA gene sequencing for species and strain-level microbiome analysis. Nat. Commun. 10:5029. 10.1038/s41467-019-13036-1 31695033 PMC6834636

[B22] Karacik B. Okay O. S. Henkelmann B. Bernhöft S. Schramm K.-W. (2009). Polycyclic aromatic hydrocarbons and effects on marine organisms in the Istanbul Strait. Environ. Int. 35, 599–606. 10.1016/j.envint.2008.11.005 19128832

[B23] King W. L. Siboni N. Kahlke T. Dove M. O'Connor W. Mahbub K. R.. (2020). Regional and oyster microenvironmental scale heterogeneity in the Pacific oyster bacterial community. FEMS Microbiol. Ecol. 96:fiaa054. 10.1093/femsec/fiaa054 32221598

[B24] Koskella B. Hall L. J. Metcalf C. J. E. (2017). The microbiome beyond the horizon of ecological and evolutionary theory. Nat. Ecol. Evol. 1, 1606–1615. 10.1038/s41559-017-0340-2 29038487

[B25] Laith A. A. Ros-Amira M. K. Sheikh H. I. Effendy A. W. M. Najiah M. (2021). Histopathological and immunological changes in green mussel, *Perna viridis*, challenged with *Vibrio alginolyticus*. Fish Shellfish Immunol. 118, 169–179. 10.1016/j.fsi.2021.08.032 34487829

[B26] Leung P. T. Y. Park T. J. Wang Y. Che C. M. Leung K. M. Y. (2014). Isoform-specific responses of metallothioneins in a marine pollution biomonitor, the green-lipped mussel *Perna viridis*, towards different stress stimulations. Proteomics 14, 1796–1807. 10.1002/pmic.201300439 24838682

[B27] Li Z. Chen R. Zuo Z. Mo Z. Yu A. (2013). Cloning, expression and identification of two glutathione S-transferase isoenzymes from *Perna viridis*. Comp. Biochem. Physiol. B. Biochem. Mol. Biol. 165, 277–285. 10.1016/j.cbpb.2013.05.005 23711756

[B28] Lin G. Lu J. Sun Z. Xie J. Huang J. Su M.. (2021). Characterization of tissue-associated bacterial community of two *Bathymodiolus* species from the adjacent cold seep and hydrothermal vent environments. Sci. Total Environ. 796:149046. 10.1016/j.scitotenv.2021.149046 34328889

[B29] Lin Z. Xu X. Xie M. Chen R. Tan Q.-G. (2021). Measuring metal uptake and loss in individual organisms: a novel ddouble stable isotope method and its application in explaining body size effects on cadmium concentration in mussels. Environ. Sci. Technol. 55, 9979–9988. 10.1021/acs.est.1c01582 34191494

[B30] Locher K. P. (2016). Mechanistic diversity in ATP-binding cassette (ABC) transporters. Nat. Struct. Mol. Biol. 23, 487–493. 10.1038/nsmb.3216 27273632

[B31] Lokmer A. Mathias Wegner K. (2015). Hemolymph microbiome of Pacific oysters in response to temperature, temperature stress and infection. ISME J. 9, 670–682. 10.1038/ismej.2014.160 25180968 PMC4331581

[B32] Martin M. (2011). Cutadapt removes adapter sequences from high-throughput sequencing reads. EMBnet J. 17:10. 10.14806/ej.17.1.200

[B33] McFall-Ngai M. J. Ruby E. G. (1991). Symbiont Recognition and Subsequent morphogenesis as early events in an animal-bacterial mutualism. Science 254, 1491–1494. 10.1126/science.1962208 1962208

[B34] Meisterhans G. Raymond N. Girault E. Lambert C. Bourrasseau L. de Montaudouin X.. (2016). Structure of Manila clam (*Ruditapes philippinarum*) microbiota at the organ scale in contrasting sets of individuals. Microb. Ecol. 71, 194–206. 10.1007/s00248-015-0662-z 26311127

[B35] Milan M. Carraro L. Fariselli P. Martino M. E. Cavalieri D. Vitali F.. (2018). Microbiota and environmental stress: how pollution affects microbial communities in Manila clams. Aq. Toxicol. 194, 195–207. 10.1016/j.aquatox.2017.11.019 29202271

[B36] Mohamed A. R. Ochsenkühn M. A. Kazlak A. M. Moustafa A. Amin S. A. (2023). The coral microbiome: towards an understanding of the molecular mechanisms of coral–microbiota interactions. FEMS Microbiol. Rev. 47:fuad005. 10.1093/femsre/fuad005 36882224 PMC10045912

[B37] Monirith I. Ueno D. Takahashi S. Nakata H. Sudaryanto A. Subramanian A.. (2003). Asia-Pacific mussel watch: monitoring contamination of persistent organochlorine compounds in coastal waters of Asian countries. Marine Pollut. Bullet. 46, 281–300. 10.1016/S0025-326X(02)00400-9 12604061

[B38] Musella M. Wathsala R. Tavella T. Rampelli S. Barone M. Palladino G.. (2020). Tissue-scale microbiota of the Mediterranean mussel (*Mytilus galloprovincialis*) and its relationship with the environment. Sci. Total Environ. 717:137209. 10.1016/j.scitotenv.2020.137209 32084687

[B39] Nalini S. Inbakandan D. Venkatnarayanan S. Mohammed Riyaz S. U. Dheenan P. S. Vinithkumar N. V.. (2019). PYRROLO isolated from marine sponge associated bacterium *Halobacillus kuroshimensis* SNSAB01 – Antifouling study based on molecular docking, diatom adhesion and mussel byssal thread inhibition. Colloids Surf. Biointerf. 173, 9–17. 10.1016/j.colsurfb.2018.09.044 30261347

[B40] Naumann M. Richter C. el-Zibdah M. Wild C. (2009). Coral mucus as an efficient trap for picoplanktonic cyanobacteria: implications for pelagic–benthic coupling in the reef ecosystem. Mar. Ecol. Prog. Ser. 385, 65–76. 10.3354/meps08073

[B41] Nyholm S. V. McFall-Ngai M. J. (2021). A lasting symbiosis: how the Hawaiian bobtail squid finds and keeps its bioluminescent bacterial partner. Nat Rev Microbiol 19, 666–679. 10.1038/s41579-021-00567-y 34089010 PMC8440403

[B42] Offret C. Paulino S. Gauthier O. Château K. Bidault A. Corporeau C.. (2020). The marine intertidal zone shapes oyster and clam digestive bacterial microbiota. FEMS Microbiol. Ecol. 96:fiaa078. 10.1093/femsec/fiaa078 32353873

[B43] Palamae S. Mittal A. Yingkajorn M. Saetang J. Buatong J. Tyagi A.. (2022). *Vibrio parahaemolyticus* isolates from Asian green mussel: molecular characteristics, virulence and their inhibition by chitooligosaccharide-tea polyphenol conjugates. Foods 11:4048. 10.3390/foods11244048 36553790 PMC9778124

[B44] Pita L. Rix L. Slaby B. M. Franke A. Hentschel U. (2018). The sponge holobiont in a changing ocean: from microbes to ecosystems. Microbiome 6:46. 10.1186/s40168-018-0428-1 29523192 PMC5845141

[B45] Prakoon W. Tunkijjanukij S. Nguyen T. T. T. Na-Nakorn U. (2010). Spatial and temporal genetic variation of green mussel, *Perna viridis* in the Gulf of Thailand and implication for aquaculture. Mar. Biotechnol. 12, 506–515. 10.1007/s10126-009-9234-x 19941027

[B46] Qin J. Li R. Raes J. Arumugam M. Burgdorf K. S. Manichanh C.. (2010). A human gut microbial gene catalogue established by metagenomic sequencing. Nature 464, 59–65. 10.1038/nature08821 20203603 PMC3779803

[B47] Rajagopal S. Nair K. V. K. Azariah J. van der Velde G. Jenner H. A. (1996). Chlorination and mussel control in the cooling conduits of a tropical coastal power station. Marine Environ. Res. 41, 201–221. 10.1016/0141-1136(95)00012-7

[B48] Rognes T. Flouri T. Nichols B. Quince C. Mah,é F. (2016). VSEARCH: a versatile open source tool for metagenomics. PeerJ 4:e2584. 10.7717/peerj.2584 27781170 PMC5075697

[B49] Spain A. M. Krumholz L. R. Elshahed M. S. (2009). Abundance, composition, diversity and novelty of soil *Proteobacteria*. The ISME J. 3, 992–1000. 10.1038/ismej.2009.43 19404326

[B50] Sweet M. J. Croquer A. Bythell J. C. (2011). Bacterial assemblages differ between compartments within the coral holobiont. Coral. Reefs 30, 39–52. 10.1007/s00338-010-0695-1

[B51] Tanca A. Abbondio M. Palomba A. Fraumene C. Manghina V. Cucca F.. (2017). Potential and active functions in the gut microbiota of a healthy human cohort. Microbiome 5:79. 10.1186/s40168-017-0293-3 28709472 PMC5513205

[B52] Tantanasarit C. Babel S. Englande A. J. Meksumpun S. (2013). Influence of size and density on filtration rate modeling and nutrient uptake by green mussel (*Perna viridis*). Mar. Pollut. Bullet. 68, 38–45. 10.1016/j.marpolbul.2012.12.027 23339878

[B53] Thomas C. Aller S. G. Beis K. Carpenter E. P. Chang G. Chen L.. (2020). Structural and functional diversity calls for a new classification of ABC transporters. FEBS Lett. 594, 3767–3775. 10.1002/1873-3468.13935 32978974 PMC8386196

[B54] Thomas T. Moitinho-Silva L. Lurgi M. Björk J. R. Easson C. Astudillo-García C.. (2016). Diversity, structure and convergent evolution of the global sponge microbiome. Nat. Commun. 7:11870. 10.1038/ncomms11870 27306690 PMC4912640

[B55] Turner L. M. Alsterberg C. Turner A. D. Girisha S. K. Rai A. Havenhand J. N.. (2016). Pathogenic marine microbes influence the effects of climate change on a commercially important tropical bivalve. Sci. Rep. 6:32413. 10.1038/srep32413 27576351 PMC5006160

[B56] Van Oppen M. J. H. Bongaerts P. Frade P. Peplow L. M. Boyd S. E. Nim H. T.. (2018). Adaptation to reef habitats through selection on the coral animal and its associated microbiome. Mol. Ecol. 27, 2956–2971. 10.1111/mec.14763 29900626

[B57] Von Byern J. Grunwald I. (2010). Biological Adhesion Systems: From Nature to Technical and Medical Application. London: Springer.

[B58] Wild C. Huettel M. Klueter A. Kremb S. G. Rasheed M. Y. M. Jørgensen B. B.. (2004). Coral mucus functions as an energy carrier and particle trap in the reef ecosystem. Nature 428, 66–70. 10.1038/nature02344 14999280

[B59] Wild C. Naumann M. Haas A. Struck U. Mayer F. Rasheed M.. (2009). Coral sand O_2_ uptake and pelagic–benthic coupling in a subtropical fringing reef, Aqaba, Red Sea. Aquat. Biol. 6, 133–142. 10.3354/ab00181

[B60] Yonge C. M. (1962). On the primitive significance of the byssus in the bivalvia and its effects in evolution. J. Mar. Biol. Ass. 42, 113–125. 10.1017/S0025315400004495

[B61] Zhou Z. Tran P. Q. Kieft K. Anantharaman K. (2020). Genome diversification in globally distributed novel marine *Proteobacteria* is linked to environmental adaptation. The ISME J. 14, 2060–2077. 10.1038/s41396-020-0669-4 32393808 PMC7367891

