# Supplementary material for: Microbiota characterization of the green mussel Perna viridis at the tissue scale and its relationship with the environment
Source: Front Microbiol. 2024 Apr 11;15:1366305. doi: 10.3389/fmicb.2024.1366305 (PMC11047130; doi:10.3389/fmicb.2024.1366305)
Supplement: Supplementary file 1 [file Table_1.docx]

**Microbiota characterization of the green mussel *Perna viridis* at the tissue scale and its relationship with the environment**

Liying Chen^1,2^, Dai Li^3^, Yawei Shen^1,2^, Zhuo Li^2^, Huanhuan Hao^2^, Caihuan Ke^1,2^, Zhang Meng^3^, Danqing Feng^2*^

*^1^ State Key Laboratory of Marine Environmental Science, College of Ocean and Earth Sciences, Xiamen University, Xiamen,China.*

*^2^ State Key Laboratory of Mariculture Breeding, College of Ocean and Earth Sciences, Xiamen University, Xiamen, China.*

*^3^ China Nuclear Power Engineering Co., Ltd, Beijing, China.*

* Corresponding author: Danqing Feng

E-mail address: dqfeng@xmu.edu.cn

**Supplementary Table S1 | Information on the sampling sites.**

| **Sample number** | **Sampling sites** | **Longitude (°)** | **Latitude (°)** |
| --- | --- | --- | --- |
| 1 | Mussel habitat | 118.33711 | 24.53543 |
| 2 | Mussel habitat | 118.34186 | 24.53529 |
| 3 | Mussel habitat | 118.34866 | 24.53558 |

**Supplementary Table S2 | Summary of sequencing reads.**

| Sample ID | Description | PE reads | Clean reads | Clean reads (%) | GC (%) |
| --- | --- | --- | --- | --- | --- |
| W1 | Seawater | 108,250 | 100,570 | 92.91 | 50.21 |
| W2 | Seawater | 60,210 | 55,830 | 92.73 | 50.53 |
| W3 | Seawater | 86,569 | 80,695 | 93.21 | 50.17 |
| S1 | Substratum | 81,461 | 74,319 | 91.23 | 51.41 |
| S2 | Substratum | 56,754 | 49,509 | 87.23 | 51.48 |
| S3 | Substratum | 46,806 | 41,279 | 88.19 | 52.57 |
| U1 | Outer shell surface | 58,521 | 51,671 | 88.29 | 51.57 |
| U2 | Outer shell surface | 64,559 | 53,024 | 82.13 | 51.92 |
| U3 | Outer shell surface | 47,493 | 40,762 | 85.83 | 52.85 |
| F1 | Foot | 77,114 | 69,007 | 89.49 | 51.46 |
| F2 | Foot | 55,423 | 51,614 | 93.13 | 50.30 |
| F3 | Foot | 78,675 | 69,165 | 87.91 | 52.05 |
| B1 | Byssus | 89,155 | 71,550 | 80.25 | 53.60 |
| B2 | Byssus | 88,652 | 72,235 | 81.48 | 53.48 |
| B3 | Byssus | 77,935 | 64,497 | 82.76 | 52.53 |
| H1 | Hemolymph | 55,883 | 52,101 | 93.23 | 50.13 |
| H2 | Hemolymph | 57,115 | 53,670 | 93.97 | 50.19 |
| H3 | Hemolymph | 49,965 | 46,616 | 93.30 | 50.21 |
| D1 | Digestive gland | 94,957 | 63,635 | 67.01 | 52.97 |
| D2 | Digestive gland | 95,628 | 65,604 | 68.60 | 53.25 |
| D3 | Digestive gland | 102,839 | 74,908 | 72.84 | 53.88 |
| G1 | Gill | 106,674 | 79,426 | 74.46 | 53.32 |
| G2 | Gill | 118,176 | 86,110 | 72.87 | 53.11 |
| G3 | Gill | 124,046 | 91,446 | 73.72 | 53.89 |
| M1 | Mantle | 132,794 | 98,018 | 73.81 | 52.56 |
| M2 | Mantle | 112,167 | 86,375 | 77.01 | 53.34 |
| M3 | Mantle | 96,162 | 75,703 | 78.72 | 53.79 |
| T1 | Testis | 109,747 | 83,883 | 76.43 | 52.05 |
| T2 | Testis | 85,570 | 56,593 | 66.14 | 52.29 |
| T3 | Testis | 70,203 | 57,441 | 81.82 | 52.98 |
| O1 | Ovary | 109,496 | 83,672 | 76.42 | 53.25 |
| O2 | Ovary | 110,553 | 84,004 | 75.99 | 52.83 |
| O3 | Ovary | 85,889 | 69,156 | 80.52 | 52.66 |

**Supplementary Table S3 | Relative abundance of the most representative microbial families in *P. viridis* tissues and environmental samples.**

| Taxon | Seawater | Substratum | Outer shell surface | Foot | Byssus | Hemolymph | Digestive gland | Gill | Mantle | Testis | Ovary |
| --- | --- | --- | --- | --- | --- | --- | --- | --- | --- | --- | --- |
| Flavobacteriaceae | 16.55 | 29.39 | 29.29 | 44.67 | 1.19 | 4.79 | 1.85 | 1.78 | 0.56 | 0.37 | 2.68 |
| Comamonadaceae | 0.02 | 2.67 | 4.00 | 0.01 | 24.17 | 0.01 | 26.16 | 50.18 | 10.45 | 8.49 | 25.63 |
| Rhodobacteraceae | 16.33 | 19.74 | 18.45 | 17.17 | 0.23 | 3.93 | 0.08 | 0.10 | 0.29 | 0.15 | 0.41 |
| Enterobacteriaceae | 0.00 | 1.06 | 1.81 | 0.00 | 6.49 | 0.00 | 12.69 | 3.85 | 25.98 | 18.99 | 2.40 |
| Spirochaetaceae | 0.00 | 0.00 | 0.01 | 0.00 | 0.00 | 59.09 | 0.00 | 0.00 | 0.05 | 0.17 | 0.01 |
| Aeromonadaceae | 0.00 | 1.28 | 2.07 | 0.00 | 1.16 | 0.00 | 27.03 | 16.25 | 2.34 | 3.90 | 2.81 |
| Weeksellaceae | 0.00 | 0.76 | 1.17 | 0.01 | 0.94 | 0.00 | 12.15 | 4.77 | 10.45 | 10.27 | 2.97 |
| Pseudomonadaceae | 0.00 | 0.34 | 0.52 | 0.01 | 4.46 | 0.00 | 5.76 | 5.37 | 5.69 | 3.10 | 7.26 |
| Chitinophagaceae | 0.00 | 0.38 | 0.55 | 0.00 | 5.54 | 0.01 | 1.03 | 2.37 | 3.43 | 1.41 | 10.63 |
| Chloroplast | 7.76 | 3.51 | 2.76 | 0.05 | 0.15 | 0.83 | 0.00 | 0.00 | 0.14 | 0.03 | 0.04 |
| Methylophilaceae | 2.67 | 0.14 | 0.23 | 0.20 | 15.54 | 0.11 | 0.01 | 0.01 | 0.06 | 0.01 | 0.03 |
| Sphingomonadaceae | 0.55 | 4.20 | 5.66 | 3.45 | 0.44 | 0.39 | 1.36 | 0.28 | 2.29 | 0.94 | 0.65 |
| SAR86_clade | 10.17 | 0.10 | 0.02 | 0.01 | 0.00 | 0.10 | 0.00 | 0.00 | 0.00 | 0.00 | 0.00 |
| Bdellovibrionaceae | 0.06 | 0.25 | 0.27 | 0.00 | 1.42 | 0.00 | 1.45 | 4.49 | 3.15 | 1.29 | 3.76 |
| Clade_I | 8.35 | 0.03 | 0.04 | 0.00 | 0.00 | 0.07 | 0.00 | 0.00 | 0.00 | 0.00 | 0.00 |
| Xanthomonadaceae | 0.00 | 0.22 | 0.31 | 0.00 | 7.14 | 0.00 | 0.19 | 0.43 | 0.85 | 0.35 | 5.27 |
| Hyphomonadaceae | 0.02 | 3.98 | 2.53 | 0.89 | 3.97 | 0.07 | 0.07 | 0.45 | 0.22 | 0.10 | 2.35 |
| Oxalobacteraceae | 0.00 | 0.22 | 0.36 | 0.00 | 0.55 | 0.00 | 0.62 | 2.57 | 0.88 | 0.53 | 8.60 |
| Saprospiraceae | 1.30 | 3.29 | 2.37 | 3.34 | 0.12 | 0.09 | 0.16 | 0.15 | 0.45 | 0.13 | 0.63 |
| Cryomorphaceae | 4.88 | 0.16 | 0.05 | 0.20 | 0.00 | 0.01 | 0.00 | 0.00 | 0.00 | 0.00 | 0.00 |
| Haliangiaceae | 0.00 | 0.11 | 0.17 | 0.01 | 5.26 | 0.00 | 0.06 | 0.20 | 3.07 | 0.39 | 1.62 |
| Microtrichaceae | 0.25 | 1.62 | 2.39 | 5.47 | 0.00 | 0.08 | 0.00 | 0.00 | 0.01 | 0.01 | 0.01 |
| Unclassified | 0.47 | 1.70 | 1.56 | 1.63 | 0.21 | 2.30 | 0.22 | 0.07 | 0.43 | 0.33 | 0.26 |
| Vibrionaceae | 0.08 | 0.45 | 0.63 | 0.03 | 0.00 | 7.98 | 0.00 | 0.00 | 0.00 | 0.00 | 0.00 |
| Acidaminococcaceae | 0.00 | 0.11 | 0.26 | 0.00 | 0.01 | 0.00 | 1.30 | 0.24 | 2.37 | 4.05 | 0.11 |
| Mycoplasmataceae | 0.00 | 0.00 | 0.09 | 0.00 | 0.00 | 8.07 | 0.00 | 0.00 | 0.00 | 0.00 | 0.00 |
| Streptococcaceae | 0.00 | 0.15 | 0.28 | 0.00 | 0.00 | 0.00 | 0.00 | 0.00 | 0.83 | 6.75 | 0.00 |
| Rubritaleaceae | 2.98 | 1.11 | 0.27 | 0.42 | 0.00 | 0.33 | 0.00 | 0.00 | 0.00 | 0.00 | 0.00 |
| Spirosomaceae | 0.00 | 0.09 | 0.12 | 0.00 | 1.76 | 0.00 | 0.50 | 1.49 | 0.54 | 0.19 | 2.87 |
| Paludibacteraceae | 0.00 | 0.10 | 0.18 | 0.00 | 0.11 | 0.00 | 0.20 | 0.13 | 1.54 | 4.42 | 0.18 |
| Halieaceae | 2.43 | 1.54 | 0.36 | 0.03 | 0.00 | 0.14 | 0.00 | 0.00 | 0.00 | 0.00 | 0.00 |
| Caulobacteraceae | 0.00 | 0.14 | 0.29 | 0.00 | 0.88 | 0.01 | 1.55 | 0.44 | 1.35 | 1.42 | 0.70 |
| SAR116 clade | 3.23 | 0.02 | 0.06 | 0.00 | 0.00 | 0.02 | 0.00 | 0.00 | 0.00 | 0.00 | 0.00 |
| Fusobacteriaceae | 0.02 | 0.00 | 0.00 | 0.00 | 0.00 | 6.38 | 0.00 | 0.00 | 0.00 | 0.00 | 0.00 |
| other Families | 21.87 | 21.13 | 20.87 | 22.39 | 18.25 | 5.18 | 5.56 | 4.37 | 22.58 | 32.22 | 18.13 |

####

####
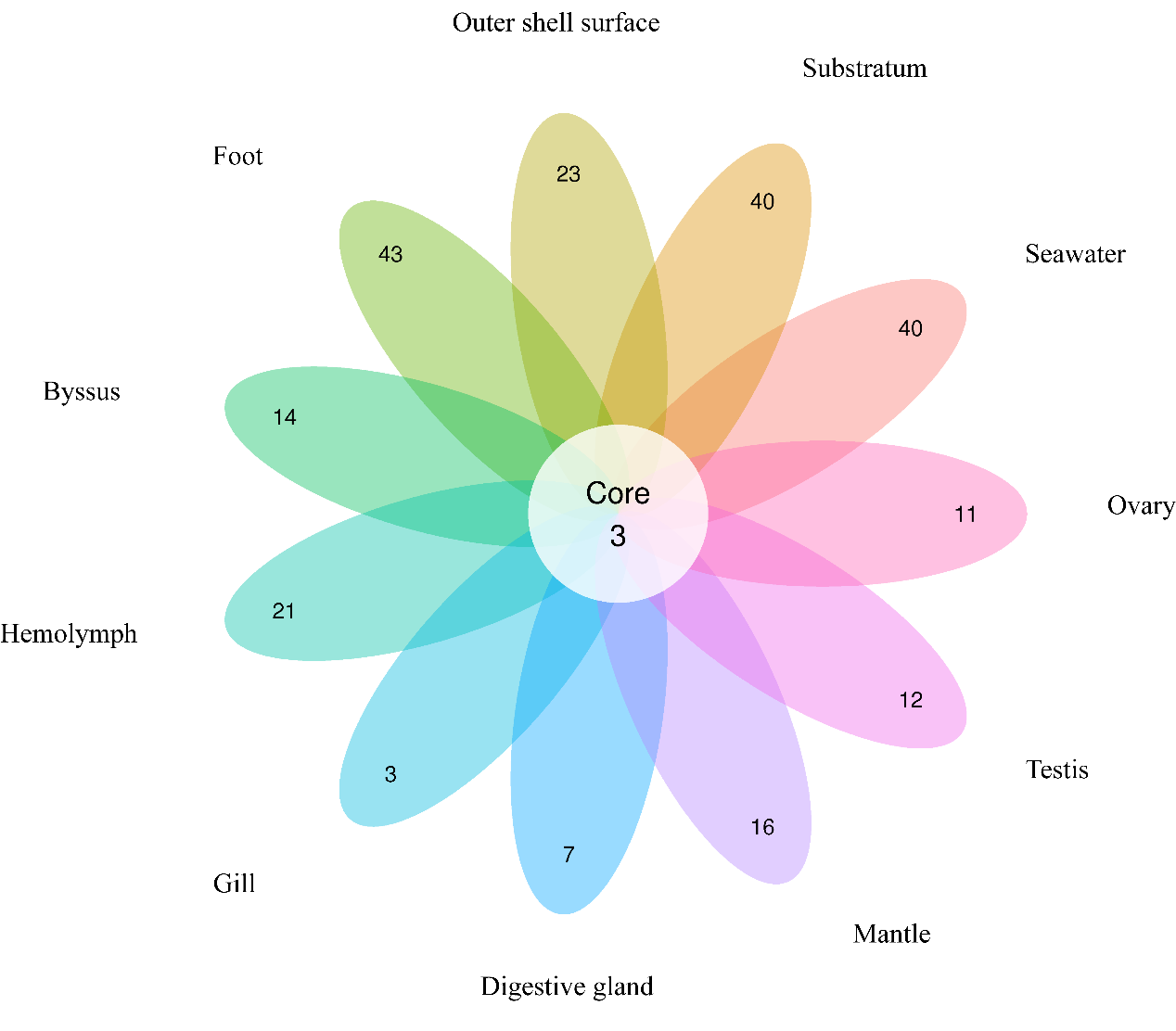


#### Supplementary Figure S1 | Petal diagram based on OTUs of samples. In the diagram, each petal represents the number of OTUs in a sample, and the white circle in the middle represents the number of overlapping OTUs in the samples.

####
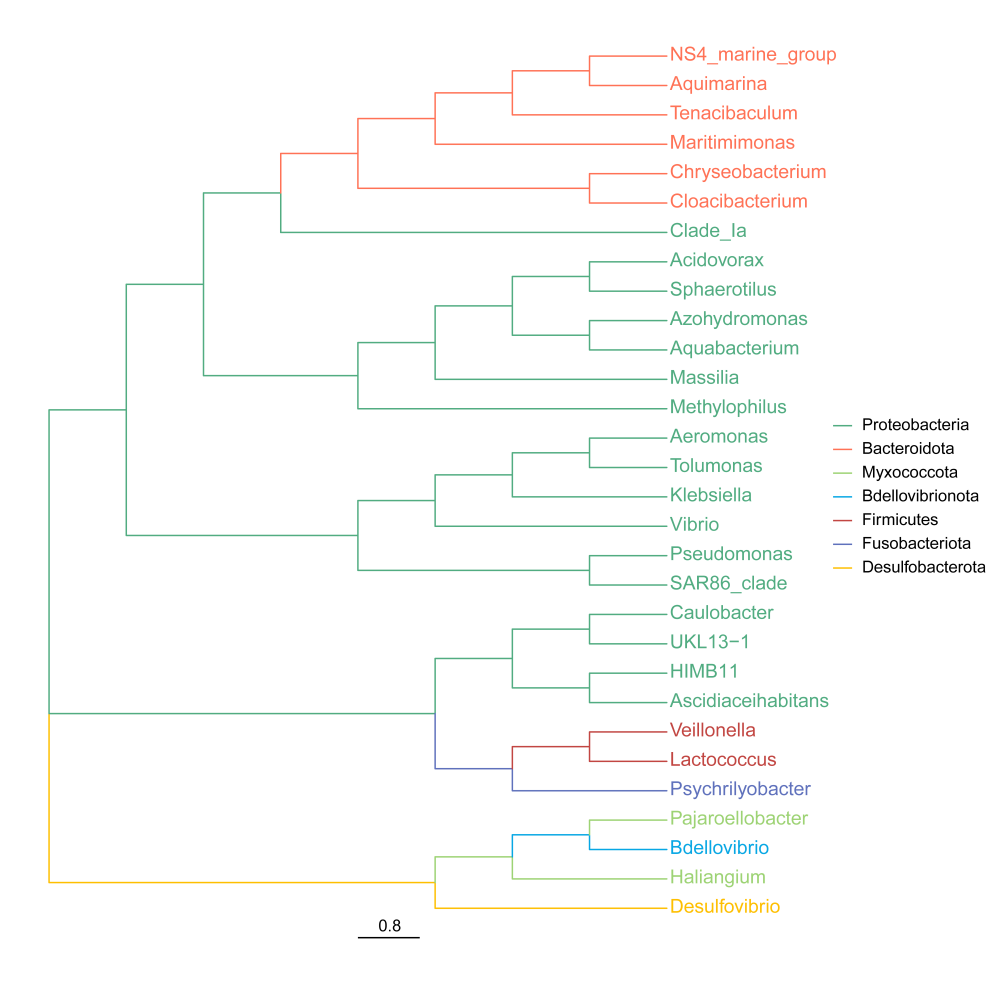


#### Supplementary Figure S2 | Phylogenetic trees of the top 30 genera in mussel tissues and environmental samples. The colors of the branches represent the corresponding phyla.
